# Evaluation of the anti-*mycobacterium tuberculosis* activity and *in vivo* acute toxicity of *Annona sylvatic*

**DOI:** 10.1186/1472-6882-14-209

**Published:** 2014-06-28

**Authors:** Rafaele CP Araujo, Francisco AR Neves, Anelise SN Formagio, Candida AL Kassuya, Maria EA Stefanello, Vanessa V Souza, Fernando R Pavan, Julio Croda

**Affiliations:** 1 Faculty of Health Sciences, Federal University of Grande Dourados, Rodovia Dourados – Itaúm. Km 12, Dourados, Mato Grosso do Sul 79804-970, Brazil; 2 Faculty of Health Sciences, University of Brasília (UNB), Brasília, Brazil; 3 Faculty of Agrarian Sciences, Federal University of Grande Dourados, Dourados, Brazil; 4 Department of Chemistry, Federal University of Paraná, Curitiba, Paraná, Brazil; 5 Biological Science Department – College of Pharmaceutical Sciences, Estadual University of São Paulo, Araraquara, Brazil

**Keywords:** *Mycobacterium tuberculosis*, *Annona sylvatica*, Resazurin reduction toxicity, Luteolin, Almunequin

## Abstract

**Background:**

The recent emergence of extensively multidrug-resistant *Mycobacterium tuberculosis* strains has further complicated the control of tuberculosis. There is an urgent need for the development of new molecular candidates antitubercular drugs. Medicinal plants have been an excellent source of leads for the development of drugs. The aim of this study was to evaluate the *in vitro* activity of 28 alcoholic extracts and essential oils of native and exotic Brazilian plants against *Mycobacterium tuberculosis* and to further study these extracts through chemical fractionation, the isolation of their constituents, and an evaluation of the *in vivo* acute toxicity of the active extracts. To the best of our knowledge this is the first chemical characterization, antituberculosis activity and acute toxicity evaluation of *Annona sylvatica*.

**Methods:**

The anti-mycobacterial activity of these extracts and their constituent compounds was evaluated using the resazurin reduction microtiter assay (REMA). To investigate the acute toxicity of these extracts *in vivo*, female Swiss mice were treated with the extracts at doses of 500, 1000 and 2000 mg · kg^-1^ of body weight. The extracts were characterized by LC-MS, and the constituents were isolated and identified by chromatographic analysis of spectroscopic data.

**Results:**

Of the 28 extracts, the methanol extract obtained from the leaves of *Annona sylvatica* showed anti-mycobacterial activity with an minimal inhibitory concentration (MIC) of 184.33 μg/mL, and the ethyl acetate fraction (EAF) resulting from liquid-liquid partitioning of the *A. sylvatica* extract showed an MIC of 115.2 μg/mL. The characterization of this extract by LC-MS identified flavonoids and acetogenins as its main constituents. The phytochemical study of the *A. sylvatica* EAF resulted in the isolation of quercetin, luteolin, and almunequin.

**Conclusions:**

Among the compounds isolated from the EAF, luteolin and almunequin were the most promising, with MICs of 236.8 μg/mL (827.28 μM) and 209.9 μg/mL (328.48 μM), respectively. The acute administration of the EAF fraction in doses of 500, 1000, and 2000 mg · kg^-1^ of body weight did not cause signs of toxicity in the treated animals.

## Background

Tuberculosis remains an important public health problem and a major cause of death worldwide; it is responsible for approximately one million deaths every year [1,2]. Due to the reduced effectiveness of current drugs resulting from the emergence of multidrug-resistant strains (MDRTB) and co-infection with HIV, there is an urgent need to develop new natural or synthetic antitubercular drugs [3-5].

The use of medicinal plants is important throughout the world, especially in traditional or alternative medicine. The search for active plant-derived compounds is a modern approach to drug discovery, especially in tropical regions with abundant flora. In underdeveloped or developing countries, medicines derived from plants are important weapons against serious diseases. Traditional medicine has enabled the treatment of common illnesses in approximately 60 to 80% of the world population [6,7]. The extraction of compounds from plants and the testing of the biological activity of those extracts and/or compounds represent the first steps toward identifying natural products or semi-synthetic derivatives that may provide new antitubercular drugs [8-12].

Research in the area of natural products has intensified, especially in the search for compounds and plant species that are active against *Mycobacterium tuberculosis* and with the development of easier, faster, and safer screening techniques [13-26]. Previous studies have demonstrated that several plants are active against *M. tuberculosis*, such as *Clavija procera* B. Ståhl, which has been shown to be active even against resistant strains [14]; *Abelmoschus esculentus* Moench; *Faurea saligna* Harv; *Parinari curatellifolia* Planch ex Benth [15]; *Maerua edulis* (Gilg & Gilg-Ben.) DeWolf; *Securidaca longepedunculata* Fres.; *Tabernaemontana elegans* Stapf; *Zanthoxylum capense* (Thunb.) Harv. [16]; *Aristolochia taliscana* Hook [17]; and *Acorus calamus* L. var. americanus [18].

According to the screening program for new antitubercular drugs developed by the National Institutes of Health of the United States, all compounds must be used in both *in vitro* and *in vivo* models to evaluate their potential as antitubercular agents [27].

This study was conducted as a preliminary *in vitro* screening of 28 plant extracts and three essential oils against *M. tuberculosis* using the REMA assay. The characterization of the active extract by LC-MS, the isolation of the main constituents of this extract, and the subsequent evaluation of the anti-*M. tuberculosis* activity and *in vivo* acute toxicity of these extracts were also performed.

## Methods

### Plant material

Different species of Asteraceae, Anacardiaceae, Annonaceae, Bignoniaceae, Euphorbiaceae, Fabaceae, Gesneriaceae, Malvaceae, Meliaceae, Myrtaceae, Rubiaceae, Sapindaceae, and Tropaeolaceae were collected in Dourados, Mato Grosso do Sul, and Maringá and Curitiba, Paraná, Brazil. The plants were identified by Dr. Armando Carlos Cervi, Dr. Maria Conceição de Souza, and Dr. Zefa Valdevina Pereira. A voucher specimen of each species was identified and deposited in the herbarium of each institution (Table 1).

**Table 1:** Plants (family and specimen), popular name, tested part (solvent), popular indication, and MICs of the extracts tested in this study

**Family**	**Specimen**	**Popular name**	**Tested part (solvent)**	**Popular indication**	**MIC (μg/mL)**
Asteraceae	*Gochnatia polymorpha* Less. UPCB 30100ª	Cambará	Bark (e)	Antitussive [28]	>500
Anacardiaceae	*Schinus terebinthifolius* Raddi. DDMS 4602^b^	Pimenta rosa, aroeirinha, Aroeira vermelha	L (m); F (hm), OE	Uterine inflammation [29]	>250
Annonaceae	*Annona crassiflora* Mart. DDMS4599^b^	Araticum do cerrado	L (m)	Antidiarrheal [30]	>250
	*Annona coriacea* Mart. DDMS186^b^		L (m)	Antidiarrheal [31]	>250
	*Annona sylvatica* St.Hill DDMS4600^b^	Araticum	L (m)	Antitussive, antipyretic, antispasmodic [32]	184.33
	*Annona cacans* Warm. DDMS	Araticum-cagão, Araticum-de-paca	L (m)		>250
	*Annona dioica* A.St.-Hill DDMS 4598^b^	Ata-rasteira, Marolo	L (m)	Antirheumatic, antidiarrheal, expectorant [33]	>250
	*Duguetia furfuracea* A.St.-Hill DDMS 166^b^	Araticum seco, Araticum-miúdo, Ata-do-mato	L (m)	Antidiarrheal, antispasmodic [34]	>250
Bignoniaceae	*Jacaranda decurrens* Cham.	Carobinha	L (m)	Astringent [35]	>250
Euphorbiaceae	*Alchornea glandulosa* Poepp. & Endl. HU7569^c^	Tamanqueiro, Tapiá, Tanheiro	L (m)	Antidiarrheal, anti-inflammatory, antirheumatic	>250
Fabaceae	*Stryphnodendron adstringens* (Mart.) Coville DDMS152^b^	Barbatimão	L (m), S (hs)	Leucorrhoea, bleeding, wound cleaning [36]	>250
Gesneriaceae	*Sinningia aggregata* (Ker. Gawl.) Wiehler		Tuber (e)		>250
	*Sinningia allagophylla* (Mart.) Wiehler MBM313530^d^	Batata-do-campo ou Batata-de-perdiz	Tuber (e)	Antipyretic, diuretic, depurative [37]	>250
	*Sinningia canescens* (Mart.) Wiehler MBM363740^d^	Rainha do abismo	Tuber (e)		>250
Malvaceae	*Hibiscus sabdariffa L.* DDMS4593^b^	Vinagreira, Azedinha	L(e), C(e)	Antispasmodic, anti-inflammatory, antioxidant, diuretic, mild laxative [38]	>250
Meliaceae	*Trichilia silvatica* DC. DDMS4662^b^	Catiguá-branco, Catiguá, Rosa-branca	L(m), S(hs), C(m), OE	Anti-inflammatory [39]	>250
Myrtaceae	*Eugenia pyriformis* Cambess. UPCB 16741ª	Uvaia	L (e)	Astringent, digestive, antitumoral, antimalarial, anti-inflammatory [40]	>250
	*Myrcia obtecta* (O. Berg) Kiaersk. var. obtecta UPCB50504^a^	Guamirim branco, Cambuí	L (e)		>250
	*Myrcia laruotteana* Camb. UPCB53303^a^	Cambuí	L (e)	Antidiarrheal [41]	>250
	*Campomanesia adamantium* (Cambess.) O.Berg DDMS^b^	Guabiroba-do-campo, Guabiroba-do-cerrado	L(m)	Antidiarrheal and anti-inflammatory [42]	>250
Rubiaceae	*Randia hebecarpa* Benth.	Limãozinho	L(m)		>250
	*Geophila repens* (L.) I.M. Johnst.	Cauá-pirí, Cauá-Pixi	L(m)		>250
	*Psychotria brachybotrya* DC.		L(e)		>250
	*Palicourea crocea* (Sw.) Roem. & Schult.		L(m)		>250
Sapindaceae	*Serjania hebecarpa* Benth.		L(m)		>250
	*Urvillea ulmaceae* Kunth.		L(m)		>250
Tropaeolaceae	*Tropaeolum majus L.*	Capuchinha	L(e), C(e), R(hs)		>250

^a.^Herbarium of the Universidade Federal do Paraná – UFPR.

^b.^Herbarium of the Faculdade de Ciências Biológicas e Ambientais – UFGD/MS.

^c.^Herbarium of the Departamento de Biologia – UEM/PR.

^d.^Herbarium of the Museu Botânico Municipal de Curitiba - PR.

L = leaves; R = root (wood + bark); S = stem (wood + bark); C = capitulum; F = fruit.

Solvents: h, hexane; hs, 90% hydroethanolic solution; m, methanol; e, ethanol.

### Preparation of extracts

The plants were air-dried at room temperature and ground with a pestle and mortar. Approximately 300 g of each sample was then exhaustively extracted by macerating the sample with 1.5 L of the appropriate solvents (Table 1) at room temperature (five times for each species at 48 h intervals). The crude extract was then isolated by the evaporation of the solvent under vacuum on a rotary evaporator. For subsequent studies, the extracts were diluted in DMSO (dimethyl sulfoxide) at 10,000 μg/mL.

### Fractionation and compound isolation

An extract that exhibited potential activity against Mycobacterium tuberculosis was dissolved in methanol:water (1:1) and partitioned with chloroform and ethyl acetate to yield the chloroform fraction (CF), ethyl acetate fraction (EAF), and hydromethanol fraction (HMF) after the evaporation of the solvents on a rotary evaporator. The resulting EAF (2.3 g) was applied to a chromatographic column on silica gel 60 (0.063-0.200 mm) (70–230 mesh) and silica flash (35-75 μm) (220–440 mesh) and eluted with a mixture of chloroform:methanol in increasing polarity, yielding 54 fractions of 10 mL each. After a thin-layer chromatography (TLC) comparison, the fractions with similar TLC patterns were grouped into ten sub-fractions. Sub-fraction 5 (173 mg) was fractionated on a Sephadex LH-20 column using H2O, H2O:MeOH 1:1, and MeOH; this process yielded two known flavonoids (6.5 mg and 5.8 mg). A series of experiments was conducted to isolate the acetogenin. Sub-fraction 8 (126 mg) was purified by flash chromatography on silica gel 60 (0.063-0.200 mm) (70–230 mesh) and silica flash (35-75 μm) (220–440 mesh) to yield almunequin (8.4 mg).

The isolated compounds were identified by an analysis of their nuclear magnetic resonance (NMR) data. NMR measurements were conducted on a Varian Mercury Plus BB spectrometer operating at 300 MHz for ^1^H and 75.5 MHz for ^13^C using CD_3_OD and CDCl_3_ as the solvents and tetramethylsilane (TMS) as the internal standard. The ^1^H NMR and ^13^C NMR data are in agreement with the reported data for quercetin [43], luteolin [44], and almunequin [32,45]:

Quercetin ^1^H NMR (300 MHz, CD_3_OD) *δ* ppm: 7.65 (1H, d, *J* = 1.8 Hz), 7.00 (1H, d, *J* = 8.9; 2.0 Hz), 6.9 (1H, d, *J* = 8.9 Hz), 6.43 (1H, d, J = 2.1 Hz), 6.20 (1H, d, J = 2.1 Hz). ^13^C NMR (75.5 MHz, CD_3_OD) *δ* ppm: 158.5, 135.6, 179.4, 163.0, 99.9, 166.2, 94.8, 159.3, 104.7, 123.5, 117.6, 145.8, 149.3, 116.5, 123.1.

Luteolin ^1^H NMR (300 MHz, CD_3_OD) *δ* ppm: 7.43 (1H, d, *J* = 2 Hz), 6.68 (1H, d, *J* = 8.4; 1.8 Hz), 6.87 (1H, d, *J* = 8.4 Hz), 6.39 (1H, d, J = 2.1 Hz), 6.20 (1H, d, J = 2.1 Hz). ^13^C NMR (75.5 MHz, CD_3_OD) *δ* ppm: 158.5, 135.6, 179.4, 163.0, 99.9, 166.2, 94.8, 159.3, 104.7, 123.5, 117.6, 145.8, 149.3, 116.5, 123.1.

Almunequin ^1^H NMR (300 MHz, CDCl_3_) *δ* ppm: 6.99 (1H, d, J = 1.2 Hz), 4.99 (1H, *dq,* J = 7.0; 1.2 Hz), 3.84 (5H, m), 3.60 (1H, m), 3.41 (2H, m), 2.25 (2H, t, J = 6.8 Hz), 2.00-1.60 (4H, m), 1.60-1.20 (m, CH_2_), 1.40 (3H, d, J = 6.9 Hz, CH_3_), 0.87 (3H, t, J = 6.8 Hz, CH_3_); ^13^C NMR (75.5 MHz, CDC1_3_): 176.4, 148.8, 134.2, 83.2, 82.1, 81.9, 79.3, 77.3, 74.4, 72.0, 71.8, 37.3, 37.0, 35.6, 32.3, 29.5, 29.3, 29.1, 28.6, 28.34, 26.1, 25.6, 31.8, 27.3, 25.1, 22.5, 22.0, 19.1, 14.0.

### Essential oil extraction

Fresh leaves of *A. sylvatica, Trichilia silvatica,* and *S. terebinthifolius* were subjected to steam distillation for 3 h using a Clevenger-type apparatus. The oil was dried by anhydrous sodium sulfate and preserved in a sealed vial at 4°C until analysis.

### LC-MS analysis of the methanolic extract of A. sylvatica

Studies using liquid chromatography coupled with mass spectrometry (LC-MS) were performed using a quad MS system spectrometer (Bruker, Bremen, Germany). Mass spectrometry was carried out in positive mode, and negative ionization (ESI) was performed using a mass/charge (m/z) ratio range from 60 to 1000. The sample was analyzed on an analytical LC system (Varian) with a ternary solvent fitted with an automatic sample, a diode array detector (PDA), and a mass spectrometer (Bruker). The LC column was a Luna C-18 column (25 cm × 4.6 mm; particle size, 5 μm) (Phenomenex, Torrance, CA, USA) with a small pre-column (2.5 cm × 3 mm) containing the same filling used to protect the analytical column. The flow rate was 1.0 mL/min, and an injected volume of 10 L was used for each analysis. All liquid chromatographic analyses were performed at 22°C. The elution was conducted using a solvent gradient of 0.1% formic acid:acetonitrile (85:15, v/v); the elution required 40 min to reach 30% formic acid and 70% acetonitrile and was then returned to the initial conditions in exactly 5 min.

### Anti-M. tuberculosis activity

The anti-*M. tuberculosis* activity of the extracts, essential oils, and compounds was determined using the REMA method [46]. *M. tuberculosis* H_37_Rv ATCC 27294 was grown for 15 days in Middlebrook 7H9 broth (Difco) supplemented with OADC enrichment (BBL/Becton-Dickinson) containing oleic acid, albumin, dextrose, and catalase; 0.5% glycerol as a carbon source; and 0.5% Tween 80 to prevent clumping. Suspensions were prepared, and the turbidity was adjusted to a McFarland no. 1 standard.

Stock solutions of the tested extracts were prepared in DMSO, and dilutions to obtain final concentrations ranging from 0.98 to 250 μg/mL were prepared in Middlebrook 7H9 broth supplemented with oleic acid, albumin, dextrose, and catalase (OADC enrichment, BBL/Becton-Dickinson, Sparks, MD, USA). Isoniazid, rifampicin, streptomycin, and ethambutol were solubilized according to the manufacturers’ recommendations (Difco Laboratories, Detroit, MI, USA) and used as positive control drugs.

After further dilutions to reach the final bacterial suspension concentration (5×10^5^ UFC/mL), 100 μL of the inoculum was added to each well of a 96-well microtiter plate containing the extracts. The assays were set up in duplicate. The plates were incubated for 7 days at 37°C, and after this incubation, 30 μL of 0.1 mg/mL resazurin was added. The wells were read for color change and fluorescence in a SPECTRAfluor Plus microfluorimeter (TECAN) (excitation/emission with 530/590 nm filters, respectively) after 24 h. The MIC (minimum inhibitory concentration) was defined as the lowest concentration resulting in a 90% growth inhibition of *M. tuberculosis*. The MIC values of isoniazid (0.06 μg/mL), rifampicin (0.03–0.06 μg/mL), streptomycin (0.25 μg/mL), and ethambutol (2.0–4.00 μg/mL) were determined in a single plate as standards [47].

A sample with an MIC value < 250 μg/mL was defined as active against *M. tuberculosis*, and further analysis was applied [48].

### Animals and acute toxicity tests

Adult female Swiss mice (19 to 24 g) from the Federal University of Grande Dourados were maintained at a controlled temperature (23°C) and humidity (50%–60%) with a constant 12 h light–dark cycle and free access to food and water. The experimental procedures were in accordance with the Ethical Principles in Animal Research and approved by the Committee for Ethics in Animal Experimentation at the Federal University of Grande Dourados (Protocol no. 005/2010). The acute toxicity studies were conducted according to OECD (Organization for Economic Cooperation and Development) Guideline 425 [33] and ANVISA (Brazilian Health Surveillance Agency) guidelines.

After 12 h of fasting, the animals were divided into four groups. The treatments were performed by single oral administration as doses of 0, 500, 1000, and 2000 mg/kg of body weight of the EAF. The animals were observed for signs of toxicity over 14 days. Behavioral parameters, mortality, the weight of the animals, and the amount of water and feed were analyzed.

After 14 days of treatment, the animals were weighed and anesthetized (ketamine and xylazine, 25 and 10 mg/kg, respectively). Blood samples were collected with and without anticoagulant (heparin sodium, Cristália). The blood samples were used to determine the hematological parameters (total and differential leukocyte count, hematocrit, hemoglobin, and erythrocyte count), and the serum samples were used for biochemical analysis (aspartate aminotransferase – AST, alanine aminotransferase – ALT, gamma glutamyl transferase – γ-GT, urea, and creatinine) [49]. The biochemical parameters were determined by spectrophotometry using Gold Analisa Diagnóstica Ltda’s kits.

Subsequently, the animals were euthanized, and the vital organs (lung, liver and right kidney) were removed and weighed (absolute and relative weight). For the histopathological analysis of these organs, the samples were fixed in 10% buffered formalin, and the tissues were processed by conventional techniques in 5-mm-thick paraffin slices. Slides were prepared and stained with hematoxylin and eosin for light microscopy examination. The evaluated parameters were reversible (degeneration) and irreversible (necrosis and apoptosis) cell damage, leukocyte infiltration, congestion, blood extravasation, and fibrosis.

The data were evaluated using an analysis of variance with an F-test, with p < 0.05 defined as significant.

## Results and discussion

### Antimycobacterial activity

The species and tested parts of the plants used in the evaluation of anti-mycobacterial activity are shown in Table 1. Of the 28 samples, only the crude extract from *A. sylvatica* (MIC = 184.33 μg/mL) exhibited promising activity (Table 1).

Several studies and screens must be performed to develop a new drug for tuberculosis; therefore, many drug discovery studies ultimately fail. A study in Mozambique screened 75 extracts of medicinal plants used for the local treatment of symptoms related to tuberculosis and identified eight extracts with moderate to significant activity against *M. tuberculosis* H37Rv. Of these extracts, six showed MICs that were higher than those observed for *A. sylvatica* and the *A. sylvatica* EAF. One extract exhibited an MIC of 62 μg/mL, and another exhibited an MIC of 15 μg/mL [16]. Another study evaluated the anti-mycobacterial activity of the crude extract of *Byrsonima crassa* (leaves and bark) and obtained an MIC value of 62.5 μg/mL for the chloroform extract of the leaves. The chloroform extract of the bark presented an MIC of 312.25 μg/mL [50].

The fractionation of the extract by partitioning in different solvents provided the chloroform (CF), ethyl acetate (EAF), and hydromethanol (HMF) fractions, which were subsequently evaluated for their anti-mycobacterial activity. An evaluation of the MIC of these fractions (Table 2) revealed the potent activity of the EAF fraction, with an MIC value of 115.2 μg/mL. The result of CIM of standard drugs, used as a control to the antimycobacterial activity tests are in agreement with the expected [47].

**Table 2:** **MIC**^**a **^**of *A. sylvatica *** **fractions and isolated compounds against *M. tuberculosis *** **using the REMA assay**

**Fraction**	**MIC (μg/mL)**	**MIC (μM)**
CF	> 250	-
EAF	115.2	-
HMF	> 250	-
Luteolin (1)	236.8	827.28
Quercetin (2)	> 250	-
Almunequin (4)	209.9	328.48
Isoniazid	0,05	
Rifampin	0,1	Rifampin
Streptomicin	0,28	Streptomicin
Ethambutol	1,88	Ethambutol

^a^:Values are means of duplicate sample.

Brazilian flora is rich in plants of the family Annonaceae, which comprises approximately 120 genera and 2000–2200 species. This family is important as a source of various edible fruits and seeds that can be used for the production of edible oils. The major components identified in members of the Annonaceae are typically acetogenins [51-53].

**Figure 1:**
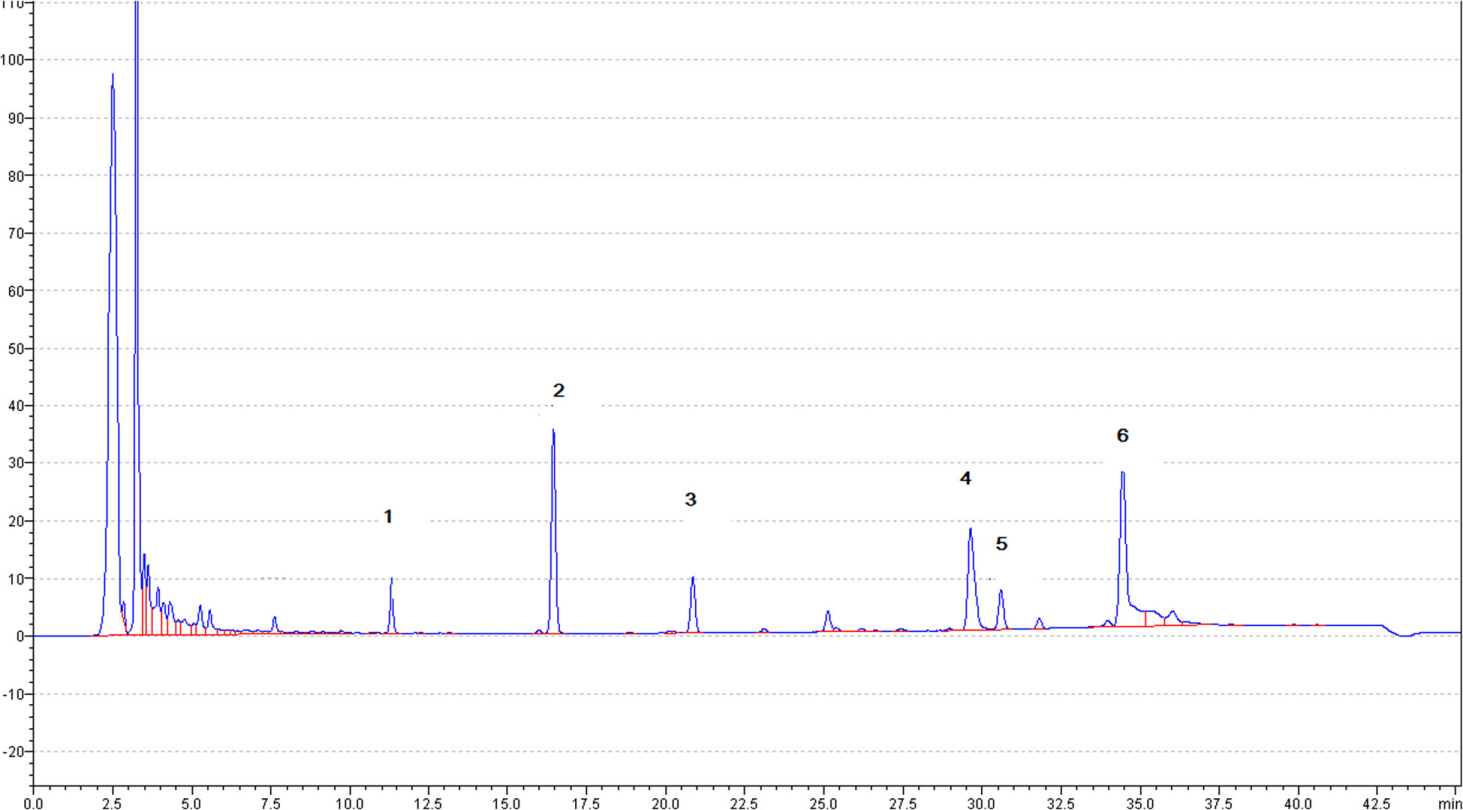
**Chromatogram of the lyophilized extract of leaves of *A. sylvatica.***

*Annona sylvatica* A.St.-Hill (formerly known as *Rollinia sylvatica* St.-Hil. Mart) is a native Brazilian plant found in Minas Gerais and São Paulo to Rio Grande do Sul. The leaves of *A. sylvatica* have been used mainly as an antipyretic in folk medicine. There are no reports in the literature on the antimicrobial activity of this species, but studies have reported that the essential oil obtained from the leaves has anti-inflammatory activity and anticancer properties [54]. A chemical study reported the isolation of sylvatin from *Rollinia sylvatica*[55]. Preliminary LC-MS studies have shown that the major metabolites in the leaves of *A. sylvatica* are flavonoids and acetogenins (Figure 1).

The results of the present study indicate that the effects of the methanolic extract of *A. sylvatica* and ethyl acetate fraction may be associated with other components of the extract (which were not evaluated) or that there might be a synergism between the active isolated compounds.

Annonaceae acetogenins are secondary metabolites derived from polyketides and are structurally characterized by a long-chain terminal α, β-unsaturated methyl γ-lactone. This hydrocarbon chain generally contains one, two, or rarely three tetrahydrofuran (THF) rings. Acetogenins belong to two different classes: bis-tetrahydrofuran non-adjacent almunequin and dihydroalmunequin 2.5 and 2, and β-hydroxy-methyl-γ-lactone and laherrandurin otivarin. The acetogenins identified in the extract of *A. sylvatica* have been reported in other *Annona*, including *A. atemoya* and *A. cherimola*[45,56-58].

In this context, the present study revealed the potential anti-*M. tuberculosis* activity of the methanolic extract of *A. sylvatica*, which is still poorly characterized in phytochemical and pharmacological terms. To characterize the possible compounds responsible for this activity, we investigated the individual compounds in the methanolic extract of *A. sylvatica*; our chromatogram of the lyophilized extract (Figure 1) showed characteristic distributions of the flavonoids luteolin (m/z 286) (1) and quercetin (m/z 302) (2) and the acetogenins laherrandurin (m/z 624) (3), almunequin (m/z 638) (4), otivarin (m/z 640) (5), and 2,5 dihydroalmunequin (m/z 640) (6) (Figure 1). This is the first report of the chemical characterization of compounds from *A. sylvatica* leaves.

The isolated compounds evaluated for anti-mycobacterial activity are shown in Table 2. In the present study, we examined the effects of two flavonoids: luteolin and quercetin. Among the isolated compounds, luteolin and almunequin showed anti-*M. tuberculosis* effects, with MIC values of 236.8 μg/mL (827.28 μM) and 209.9 μg/mL (328.48 μM), respectively. Our data indicate that quercetin, unlike luteolin and almunequin, failed to exhibit anti-mycobacterial activity at low concentrations.

Luteolin isolated from the flowers of *Chromolaena odorata* showed weak activity (699.3 μM MIC) against *M. tuberculosis*[28]. However, luteolin isolated from *Ficus chlamydocarpa* showed stronger activity against *M. tuberculosis* and *M. smegmatis* (78.12 μg/mL MIC) [29]. Nevertheless, the luteolin isolated from the whole plant of *Gentianopsis paludosa* was inactive against these microorganisms [30]. Having established the inhibitory effects of luteolin on *M. tuberculosis,* we investigated whether this agent exhibits a structure-activity relationship in terms of its biological function. The isolated flavonoids belong to the flavone (luteolin) and flavon-3-ol (quercetin) classes. The structures of quercetin and luteolin share double bonds between C2 and C3 in ring C, the 3^’^, 4’ -diOH ring A groups, and the 7, 8 -diOH ring A groups. However, ring C in these compounds shows considerable variation, as it is a 3-OH group in quercetin. This variation shows that catechol groups could not confer appreciable activity when they were found on both ring A and ring B. From a structural point of view, this finding suggests that the 3-OH in quercetin results in an inactivation of the structure, which might be critical for *M. tuberculosis* survival. The observed results for quercetin are consistent with those observed for quercetin isolated from *Helichrysum melanacme* against *M. tuberculosis*[31]. Based on our data and those of others [31], we suggest that the absence of hydroxylation in the C3 in structure of the luteolin is required for anti-mycobacterial activity. These results may provide a basis for the further design of new anti-mycobacterial drugs.

The compound almunequin was very difficult to isolate. Almunequin is a C37 annonaceous acetogenin with a bis-tetrahydrofuranic structure containing hydroxyl groups and α, β-unsaturated γ-lactone methyl group. The structure-activity relationship for almunequin can be attributed the α, β-unsaturated lactone present at the long-chain terminal. The α, β-unsaturated lactones are a class of synthetic and naturally occurring compounds that exhibit a large spectrum of important pharmacological properties [34-38,59].

### Acute toxicity

The EAF fraction of *A. sylvatica* produced significant weight gain in treated animals (Table 3). During treatment, no clinical signs of toxicity were observed, and no death was recorded. There were also no changes in food or water intake. The oral administration of this fraction generally did not produce toxic effects on the behavior of adult female Swiss mice. Apart from the weight increase, no visible clinical signs of toxicity were observed.

**Table 3:** Body weight and relative weights of organs of animals exposed to EAF fraction

**Parameters**	**Control**	**500 mg kg**^**-1**^	**1000 mg kg**^**-1**^	**2000 mg kg**^**-1**^	p*
Corporeal weight	21.09 ± 1.31	23.24 ± 1.71	22.24 ± 0.64	23.08 ± 0.74	0.0205
Liver	6.31 ± 0.45	5.98 ± 0.32	5.96 ± 0.70	5.89 ± 0.55	0.5294
Lungs	0.64 ± 0.06	0.56 ± 0.03	0.67 ± 0.15	0.68 ± 0.07	0.1458
Heart	0.47 ± 0.06	0.42 ± 0.02	0.43 ± 0.03	0.48 ± 0.03	0.0666
Right kidney	0.53 ± 0.05	0.58 ± 0.02	0.57 ± 0.05	0.57 ± 0.02	0.1773
Left kidney	0.56 ± 0.03	0.56 ± 0.02	0.59 ± 0.04	0.54 ± 0.08	0.7158
Spleen	0.53 ± 0.19	0.43 ± 0.02	0.48 ± 0.03	0.46 ± 0.04	0.5074

Relative weight (%) of the liver, lung, heart, right kidney, left kidney and spleen of female Swiss mice treated with 500, 1000 and 2000 mg kg^-1^ of the EAF fraction of *A. sylvatica*. Data are expressed as the mean ± standard deviation SISVAR 5.3 [25]. (n=6) is the number of animals in each group. p*=5%.

There was no evidence for differences in physiological or behavioral responses between the control group and any of the treated groups at any time. There were also no differences in the consumption of food and water.

The hematologic parameters of the treated groups did not differ from those of the control group. A biochemical evaluation confirmed these results; there were no significant differences in the AST, ALT, or γ GT results between the control group and treated animals. These enzymes are liver function markers, and changes in these parameters may result from reversible or irreversible hepatocellular membrane damage. Changes in these markers are often associated with necrosis, cholestasis, hypoxia, hypoperfusion, inflammation, infectious agents and toxins, or excess lipid or glycogen deposition in hepatocytes [39]. The integrity of liver function was assessed by histopathological analysis of the liver, and we found no damage associated with hepatotoxicity. The effects of the acute administration of the EAF fraction of *A. sylvatica* on hematological and biochemical parameters are presented in Table 4.

**Table 4:** **Biochemical and hematological parameters of Swiss mice exposed to the EAF fraction of *A. sylvatica *** **in the acute toxicity study**

**Parameters**	**Control**	**500 mg kg**^**-1**^	**1000 mg kg**^**-1**^	**2000 mg kg**^**-1**^	p*
Creatinine (mg/dL)	0.29 ± 0.03	0.29 ± 0.07	0.28 ± 0.06	0.28 ± 0.05	0.8100
Urea (mg/dL)	43.66 ± 4.27	44.33 ± 4.41	45 ± 5.03	42.5 ± 4.23	0.6391
AST (U/L)	76 ± 4.45	78 ± 5.91	77 ± 4.94	79 ± 4.99	0.9015
ALT (U/L	52.5 ± 4.27	48.5 ± 4.37	50 ± 4.27	53 ± 4.75	0.1990
γ GT (U/L)	11 ± 1.41	11.5 ± 1.37	11 ± 1.26	12 ± 12.6	0.5218
RBC (x 106/mm3)	7.06 ± 0.12	7.11 ± 0.11	7.1 ± 0.10	7.15 ± 0.08	0.6161
HT (%)	45.33 ± 3.66	44.68 ± 3.55	44.5 ± 2.81	45.16 ± 3.9	0.9721
WBC (%)	8.16 ± 0.75	8.33 ± 1.03	8 ± 0.63	8.33 ± 0.81	0.4138
PLT (x 103/μL)	916 ± 10.78	928 ± 9.77	928.66 ± 27	925.16 ± 13	0.5353
LYNF (%)	77 ± 1.63	71±1.36	77.83 ± 0.89	79 ± 2.06	0.4913
NEUT (%)	19.16 ± 1.02	20 ± 1.69	19 ± 0.13	17 ± 1.14	0.5783

Hematological and biochemical parameters of female Swiss mice treated acutely orally with vehicle (control) or the EAF fraction of *A. sylvatica* at doses of 500 mg kg^-1^ 1000 mg kg^-1^ and 2000 mg kg-^1^ Values represent the mean ± SD. (n=6) is the number of animals in each group. p*=5%.

AST = Aspartate aminotransferase.

ALT = Alanine aminotransferase.

γ GT = Gamma glutamyl transferase.

RBC = Red Blood Cell.

HT = Hematocrite.

WBC = White Blood Cell.

PLT= Platelets.

LYNF = Lymphocytes.

NEUT = Neutrophils.

**Figure 2:**
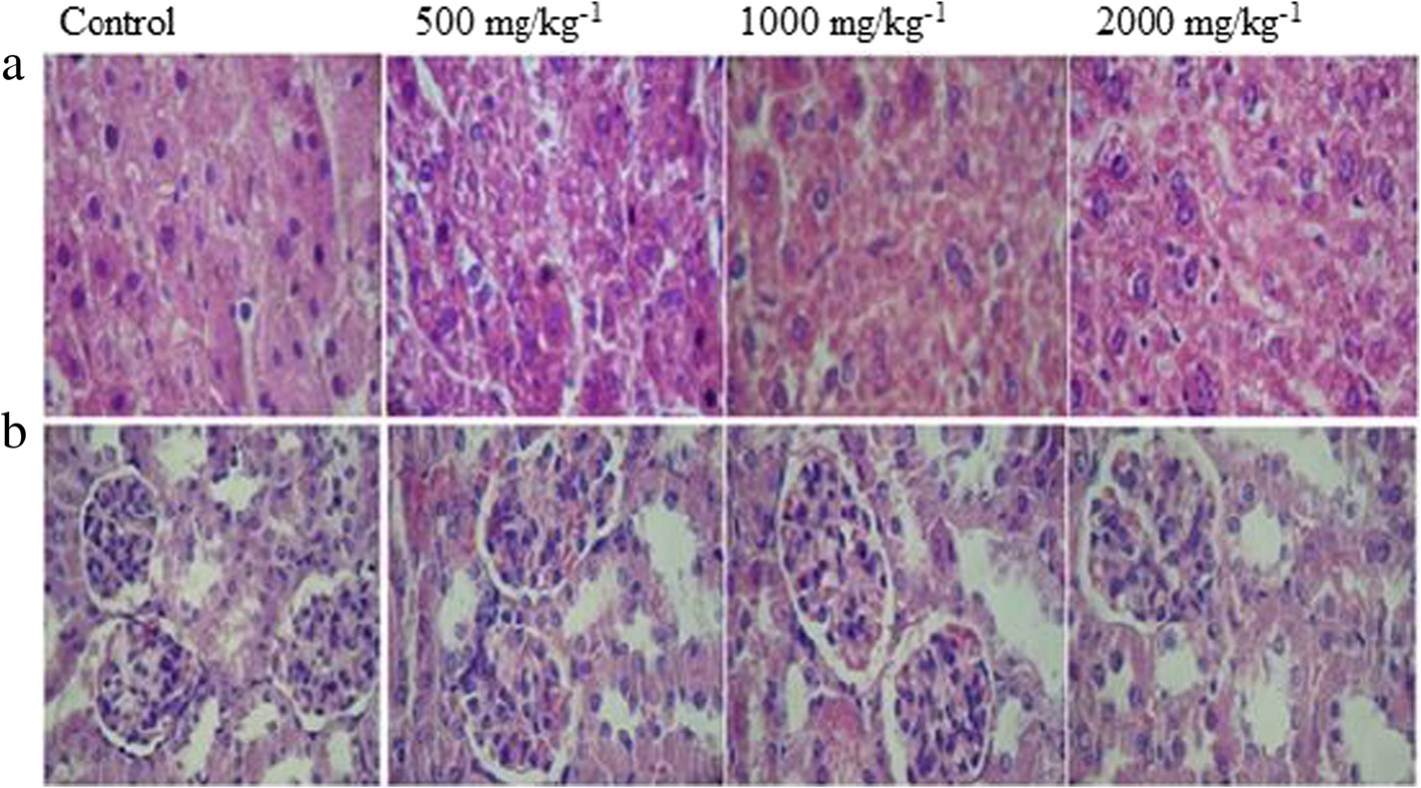
**Histological analysis of acute toxicity at doses of 500, 1000 and 2000 mg/kg**^**-1 **^**of EAF fraction (hematoxylin – eosin staining, 100X magnification). a)** Longitudinal section of liver; **b)** longitudinal section of kidney.

The EAF fraction of *A. sylvatica* had no acute toxicity, as evidenced by the absence of relevant clinical signs in the toxicological screening and the absence of death throughout the observation period. Hippocratic screening such as this is often used for the preliminary screening of plants for toxicological and pharmacological activity. The EAF fraction also did not have any influence on the consciousness of the animals during the observed period. No effects on the motor coordination or reflexes of the treated animals were observed. At doses of 500, 1000, and 2000 mg/kg, the EAF fraction produced no dose-dependent changes in histopathology between the control group and the treated animals (Figure 2). Therefore, EAF has low toxicity at high doses in the short term. Additional studies are required to determine the safety of this fraction over a prolonged period.

The acute administration of the EAF produced no toxic effects in adult female Swiss mice. No visible clinical signs of toxicity, such as irritability, twisting, righting reflex, tremors, convulsions, breathing, weight loss, or death, were observed. In the present study, we did not observe an increase in the accumulation of urea or creatinine. These results were also confirmed by histopathological analysis of the kidneys, which indicates that the acute oral administration of *A. sylvatica* does not induce nephrotoxicity.

### Ethics statement

These field studies did not involve endangered or protected species and no specific permits were required for the described studies. The studies performed with species of Annonaceae, Bignonaceae, Meliaceae, Fabaceae and Myrcia species were collected in particular area, with access permitted the researchers to collect botanical material. The species of Asteraceae, Anacardiaceae, Tropaeolaceae and Malvaceae were collected in the Medicinal Plants Garden of the Federal University of Grande Dourados, and Gesneriaceae in the Municipal Botanical Museum of Curitiba. The Rubiaceae were collected in a Brazilian stretch of the Upper Paraná River, Porto Rico, park ecosystem components collection for scientific purposes. The work with Sinningia species had an access authorization to genetic patrimony given by National Research Council (CNPq, 010087/2012-5). Professor Armando C. Cervi (Federal University of Paraná) collected *S. aggregata* and *S. canescens*, and Clarice Bolfe Poliquesi (Municipal Botanical Museum of Curitiba) collected *Sinningia allagophylla*.

## Conclusion

To the best of our knowledge, this is the first chemical characterization, evaluation of antitubercular activity, and analysis of the *in vivo* acute toxicity of the *A. sylvatica* methanolic extract. Our study demonstrates the potential anti-mycobacterial activity and the lack of *in vivo* acute toxicity of its isolated compounds.

## Competing interests

The authors declare that they have no competing interests.

## Authors’ contribution

RCP and VVS performed the experimental work and data analyses of the antimycobacterial activity tests and the *in vivo* acute toxicity tests, respectively, and wrote the manuscript. JC designed the study and FRP supervised experimental work. CALK and ASNF performed the plant extractions and LC-MS and NMR experimental work. JC, FRP, FARN e MEAS have reviewed the manuscript. All authors read and approved the final manuscript.

## Pre-publication history

The pre-publication history for this paper can be accessed here:

http://www.biomedcentral.com/1472-6882/14/209/prepub

## References

[B1] Organization WH Health Systems Profile 2006 Somalia. Geneva: WHO

[B2] Plague of poverty? The World Health Organization, Tuberculosis and International Development 1945–1980

[B3] Tripathi RP Tewari N Dwivedi N Tiwari VK Fighting tuberculosis: an old disease with new challenges Med Res Rev 2005 25 93 131 15389729 10.1002/med.20017

[B4] Espinal MA The global situation of MDR-TB Tuberculosis (Edinb) 2003 83 44 51 12758188 10.1016/s1472-9792(02)00058-6

[B5] Copp BR Pearce AN Natural product growth inhibitors of Mycobacterium tuberculosis Nat Prod Rep 2007 24 278 297 17389998 10.1039/b513520f

[B6] Zar HJ Udwadia ZF Advances in tuberculosis 2011–2012 Thorax 2013 68 283 287 23321600 10.1136/thoraxjnl-2012-203127

[B7] Chen JJ Yang CS Peng CF Chen IS Miaw CL Dihydroagarofuranoid sesquiterpenes, a lignan derivative, a benzenoid, and antitubercular constituents from the stem of Microtropis japonica J Nat Prod 2008 71 1016 1021 18471021 10.1021/np800097t

[B8] Cooper EL Drug Discovery, CAM and Natural Products Evid Based Complement Alternat Med 2004 1 215 217 15841253 10.1093/ecam/neh032 PMC538505

[B9] Yang PS Cheng MJ Peng CF Chen JJ Chen IS Endiandric Acid Analogues from the Roots of Beilschmiedia erythrophloia J Nat Prod 2009 72 53 58 19072217 10.1021/np800504w

[B10] Molina-Salinas GM Borquez J Ardiles A Said-Fernandez S Loyola LA San-Martin A Gonzalez-Collado I Pena-Rodriguez LM Antituberculosis activity of natural and semisynthetic azorellane and mulinane diterpenoids Fitoterapia 2010 81 50 54 19635530 10.1016/j.fitote.2009.07.005

[B11] Molina-Salinas GM Borquez J Said-Fernandez S Loyola LA Yam-Puc A Becerril-Montes P Escalante-Erosa F Pena-Rodriguez LM Antituberculosis activity of alkylated mulinane diterpenoids Fitoterapia 2010 81 219 222 19781604 10.1016/j.fitote.2009.09.006

[B12] Lekphrom R Kanokmedhakul S Kanokmedhakul K Bioactive diterpenes from the aerial parts of Anisochilus harmandii Planta Med 2010 76 726 728 19950052 10.1055/s-0029-1240656

[B13] Rajabi L Courreges C Montoya J Aguilera RJ Primm TP Acetophenones with selective antimycobacterial activity Lett Appl Microbiol 2005 40 212 217 15715647 10.1111/j.1472-765X.2005.01657.x

[B14] Rojas R Caviedes L Aponte JC Vaisberg AJ Lewis WH Lamas G Sarasara C Gilman RH Hammond GB Aegicerin, the first oleanane triterpene with wide-ranging antimycobacterial activity, isolated from Clavija procera J Nat Prod 2006 69 845 846 16724857 10.1021/np050554l PMC5507661

[B15] Chimponda T Mukanganyama S Antimycobacterial activities of selected medicinal plants from Zimbabwe against Mycobacterium aurum and Corynebacterium glutamicum Trop Biomed 2010 27 595 610 21399602

[B16] Luo X Pires D Ainsa JA Gracia B Mulhovo S Duarte A Anes E Ferreira MJ Antimycobacterial evaluation and preliminary phytochemical investigation of selected medicinal plants traditionally used in Mozambique J Ethnopharmacol 2011 137 114 120 21571059 10.1016/j.jep.2011.04.062

[B17] Leon-Diaz R Meckes M Said-Fernandez S Molina-Salinas GM Vargas-Villarreal J Torres J Luna-Herrera J Jimenez-Arellanes A Antimycobacterial neolignans isolated from Aristolochia taliscana Mem Inst Oswaldo Cruz 2010 105 45 51 20209328 10.1590/s0074-02762010000100006

[B18] Webster D Lee TD Moore J Manning T Kunimoto D LeBlanc D Johnson JA Gray CA Antimycobacterial screening of traditional medicinal plants using the microplate resazurin assay Can J Microbiol 2010 56 487 494 20657619 10.1139/w10-035

[B19] Jimenez-Arellanes A Leon-Diaz R Meckes M Tapia A Molina-Salinas GM Luna-Herrera J Yepez-Mulia L Antiprotozoal and Antimycobacterial Activities of Pure Compounds from Aristolochia elegans Rhizomes Evid Based Complement Alternat Med 2012 2012 593403 22454670 10.1155/2012/593403 PMC3292206

[B20] Leon-Diaz R Meckes-Fischer M Valdovinos-Martinez L Campos MG Hernandez-Pando R Jimenez-Arellanes MA Antitubercular activity and the subacute toxicity of (-)-Licarin A in BALB/c mice: a neolignan isolated from Aristolochia taliscana Arch Med Res 2013 44 99 104 23291382 10.1016/j.arcmed.2012.12.006

[B21] Rojas R Bustamante B Ventosilla P Fernadez I Caviedes L Gilman RH Lock O Hammond GB Larvicidal, antimycobacterial and antifungal compounds from the bark of the Peruvian plant Swartzia polyphylla DC Chem Pharm Bull (Tokyo) 2006 54 278 279 16462085 10.1248/cpb.54.278

[B22] Tran T Saheba E Arcerio AV Chavez V Li QY Martinez LE Primm TP Quinones as antimycobacterial agents Bioorg Med Chem 2004 12 4809 4813 15336259 10.1016/j.bmc.2004.07.015

[B23] Rijo P Simoes MF Francisco AP Rojas R Gilman RH Vaisberg AJ Rodriguez B Moiteiro C Antimycobacterial metabolites from Plectranthus: royleanone derivatives against Mycobacterium tuberculosis strains Chem Biodivers 2010 7 922 932 20397225 10.1002/cbdv.200900099

[B24] Chen JJ Wu HM Peng CF Chen IS Chu SD: **seco-Abietane diterpenoids, a phenylethanoid derivative, and antitubercular constituents from Callicarpa pilosissima** J Nat Prod 2009 72 223 228 19193025 10.1021/np800721f

[B25] Scher JM Schinkovitz A Zapp J Wang Y Franzblau SG Becker H Lankin DC Pauli GF Structure and anti-TB activity of trachylobanes from the liverwort Jungermannia exsertifolia ssp. cordifolia J Nat Prod 2010 73 656 663 20353194 10.1021/np900806j

[B26] Truong NB Pham CV Doan HT Nguyen HV Nguyen CM Nguyen HT Zhang HJ Fong HH Franzblau SG Soejarto DD Chau MV Antituberculosis cycloartane triterpenoids from Radermachera boniana J Nat Prod 2011 74 1318 1322 21469696 10.1021/np200022b PMC3703769

[B27] Orme I Search for new drugs for treatment of tuberculosis Antimicrob Agents Chemother 2001 45 1943 1946 11408205 10.1128/AAC.45.7.1943-1946.2001 PMC90582

[B28] Suksamrarn A Chotipong A Suavansri T Boongird S Timsuksai P Vimuttipong S Chuaynugul A Antimycobacterial activity and cytotoxicity of flavonoids from the flowers of Chromolaena odorata Arch Pharm Res 2004 27 507 511 15202555 10.1007/BF02980123

[B29] Kuete V Ngameni B Simo CC Tankeu RK Ngadjui BT Meyer JJ Lall N Kuiate JR Antimicrobial activity of the crude extracts and compounds from Ficus chlamydocarpa and Ficus cordata (Moraceae) J Ethnopharmacol 2008 120 17 24 18718518 10.1016/j.jep.2008.07.026

[B30] Yeung MF Lau CB Chan RC Zong Y Che CT Search for antimycobacterial constituents from a Tibetan medicinal plant, Gentianopsis paludosa Phytother Res 2009 23 123 125 19107824 10.1002/ptr.2506

[B31] Lall N Hussein AA Meyer JJ Antiviral and antituberculous activity of Helichrysum melanacme constituents Fitoterapia 2006 77 230 232 16529879 10.1016/j.fitote.2006.01.007

[B32] Fujimoto Y Murasaki C Shimada H Nishioka S Kakinuma K Singh S Singh M Gupta YK NMR data of Almunequin Chem Pharm Bull 1994 36 48023246042

[B33] OECD OfEC-oaD-OECD Guideline 425: Acute Oral Toxicity: Up-and-Down Procedure Book OECD Guideline 425: Acute Oral Toxicity: Up-and-Down Procedure (Editor ed.^eds.) 2008 City

[B34] Fatima A Kohn LK Carvalho JE Pilli RA Cytotoxic activity of (S)-goniothalamin and analogues against human cancer cells Bioorg Med Chem 2006 14 622 631 16202605 10.1016/j.bmc.2005.08.036

[B35] Albrecht A Morana F Fraile A Jorgensen KA Organophosphorus reagents in organocatalysis: synthesis of optically active alpha-methylene-delta-lactones and delta-lactams Chemistry 2012 18 10348 10354 22706879 10.1002/chem.201201325

[B36] Lapalikar GV Taylor MC Warden AC Scott C Russell RJ Oakeshott JG F420H2-dependent degradation of aflatoxin and other furanocoumarins is widespread throughout the actinomycetales PLoS One 2012 7 e30114 22383957 10.1371/journal.pone.0030114 PMC3288000

[B37] Tormo JR Estornell E Gallardo T Gonzalez MC Cave A Granell S Cortes D Zafra-Polo MC Gamma-lactone-Functionalized antitumoral acetogenins are the most potent inhibitors of mitochondrial complex I Bioorg Med Chem Lett 2001 11 681 684 11266168 10.1016/s0960-894x(01)00036-1

[B38] Gallardo T Zafra-Polo MC Tormo JR Gonzalez MC Franck X Estornell E Cortes D Semisynthesis of antitumoral acetogenins: SAR of functionalized alkyl-chain bis-tetrahydrofuranic acetogenins, specific inhibitors of mitochondrial complex I J Med Chem 2000 43 4793 4800 11123988 10.1021/jm000911j

[B39] Ezeja MI Anaga AO Asuzu IU Acute and sub-chronic toxicity profile of methanol leaf extract of Gouania longipetala in rats J Ethnopharmacol 2014 151 1155 1164 24384377 10.1016/j.jep.2013.12.034

[B40] Chavasco JM Prado EFBH Cerdeira CD Leandro FD Coelho LF Silva JJ Chavasco JK Dias AL Evaluation of antimicrobial and cytotoxic activities of plant extracts from southern Minas Gerais cerrado Rev Inst Med Trop Sao Paulo 2014 56 13 20 24553603 10.1590/S0036-46652014000100002 PMC4085825

[B41] Salvador MJ de Lourenco CC Andreazza NL Pascoal AC Stefanello ME Antioxidant capacity and phenolic content of four Myrtaceae plants of the south of Brazil Nat Prod Commun 2011 6 977 982 21834237

[B42] Pascoal AC Ehrenfried CA Lopez BG de Araujo TM Pascoal VD Gilioli R Anhe GF Ruiz AL Carvalho JE Stefanello ME Salvador MJ Antiproliferative activity and induction of apoptosis in PC-3 cells by the chalcone cardamonin from Campomanesia adamantium (Myrtaceae) in a bioactivity-guided study Molecules 2014 19 1843 1855 24514747 10.3390/molecules19021843 PMC6271740

[B43] Ross SA ElSohly MA Sultana GN Mehmedic Z Hossain CF Chandra S Flavonoid glycosides and cannabinoids from the pollen of Cannabis sativa L Phytochem Anal 2005 16 45 48 15688956 10.1002/pca.809

[B44] Silva DA Silva TMS Lins ACS Costa DA Cavalcante JMS Matias WN Souza MFV Braz Filho R Constituintes químicos e atividade antioxidante de Sida galheirensiS Ulbr. (Malvaceae) Quím Nova 2006 29 1250 1254

[B45] Cortes DMS Dupont B Davoust D Bioactive acetogenins from seeds of Annona cherimolia Phytochemistry 1993 32 1475 1482

[B46] Palomino JC Martin A Camacho M Guerra H Swings J Portaels F Resazurin microtiter assay plate: simple and inexpensive method for detection of drug resistance in Mycobacterium tuberculosis Antimicrob Agents Chemother 2002 46 2720 2722 12121966 10.1128/AAC.46.8.2720-2722.2002 PMC127336

[B47] Collins L Franzblau SG Microplate alamar blue assay versus BACTEC 460 system for high-throughput screening of compounds against Mycobacterium tuberculosis and Mycobacterium avium Antimicrob Agents Chemother 1997 41 1004 1009 9145860 10.1128/aac.41.5.1004 PMC163841

[B48] Gu JQ Wang Y Franzblau SG Montenegro G Yang D Timmermann BN Antitubercular Constituents of Valeriana laxiflora Planta Med 2004 70 509 514 15229801 10.1055/s-2004-827149

[B49] Balani T Agrawal S Thaker AM Hematological and biochemical changes due to short-term oral administration of imidacloprid Toxicol Int 2011 18 2 4 21430911 10.4103/0971-6580.75843 PMC3052578

[B50] Higuchi CT Pavan FR Leite CQF Triterpenes and antitubercular activity of Byrsonima crassa Quim Nova 2008 31 1719 1721

[B51] Zeng L Wu FE Oberlies NH McLaughlin JL Sastrodihadjo S Five new monotetrahydrofuran ring acetogenins from the leaves of Annona muricata J Nat Prod 1996 59 1035 1042 8946744 10.1021/np960447e

[B52] Fatope MO Audu OT Takeda Y Zeng L Shi G Shimada H McLaughlin JL Bioactive ent-kaurene diterpenoids from Annona senegalensis J Nat Prod 1996 59 301 303 8882434 10.1021/np9601566

[B53] Liu XX Pilarinou E McLaughlin JL Two novel acetogenins, annoglaxin and 27-hydroxybullatacin, from Annona glabra J Nat Prod 1999 62 848 852 10395501 10.1021/np980552j

[B54] Formagio AS Vieira Mdo C, Dos Santos LA, Cardoso CA, Foglio MA, de Carvalho JE, Andrade-Silva M, Kassuya CA: **Composition and evaluation of the anti-inflammatory and anticancer activities of the essential oil from Annona sylvatica A. St.-Hil** J Med Food 2013 16 20 25 23297712 10.1089/jmf.2011.0303

[B55] Mikolajczak KJ Madrigal RV Rupprecht JK Hui YH Liu YM Smith DL McLaughlin JL Sylvaticin: a new cytotoxic and insecticidal acetogenin from Rollinia sylvatica (Annonaceae) Experientia 1990 46 10.1007/BF01951779 2178957

[B56] Wu P Chen WS Hu TS Yao ZJ Wu YL Atemoyacin E, a bis-tetrahydrofuran annonaceous acetogenin from Annona atemoya seeds J Asian Nat Prod Res 2001 3 177 182 11491392 10.1080/10286020108041388

[B57] Duret PHR Cavé A Annonisin, a bis-tetrahydrofuran acetogenin from Annona atemoya seeds Phytochemistry 1997 45 1423 1426

[B58] Formagio AS Kassuya CA Neto FF Volobuff CR Iriguchi EK Vieira Mdo C Foglio MA The flavonoid content and antiproliferative, hypoglycaemic, anti-inflammatory and free radical scavenging activities of Annona dioica St. Hill BMC Complement Altern Med 2013 13 14 23311341 10.1186/1472-6882-13-14 PMC3551637

[B59] Le Goff G Martin MT Servy C Cortial S Lopes P Bialecki A Smadja J Ouazzani J Isolation and characterization of alpha, beta-unsaturated gamma-lactono-hydrazides from Streptomyces sp J Nat Prod 2012 75 915 919 22591466 10.1021/np300026p

